# Vocabulary at the Living–Machine Interface: A Narrative Review of Shared Lexicon for Hybrid AI

**DOI:** 10.3390/biomimetics10110723

**Published:** 2025-10-29

**Authors:** Andrew Prahl, Yan Li

**Affiliations:** 1 Wee Kim Wee School of Communication & Information, Nanyang Technological University, Singapore 637718, Singapore; 2 Campus for Research Excellence and Technological Enterprise, Singapore 138602, Singapore

**Keywords:** Hybrid-AI, human–machine systems, biohybrids, conceptualization, concept-mapping

## Abstract

The rapid rise of bio-hybrid robots and hybrid human–AI systems has triggered an explosion of terminology that inhibits clarity and progress. To investigate how terms are defined, we conduct a narrative scoping review and concept analysis. We extract 60 verbatim definitions spanning engineering, human–computer interaction, human factors, biomimetics, philosophy, and policy. Entries are coded on three axes: agency locus (human, shared, machine), integration depth (loose, moderate, high), and normative valence (negative, neutral, positive), and then clustered. Four categories emerged from the analysis: (i) machine-led, low-integration architectures such as neuro-symbolic or “Hybrid-AI” models; (ii) shared, moderately integrated systems like mixed-initiative cobots; (iii) human-led, medium-coupling decision aids; and (iv) human-centric, low-integration frameworks that focus on user agency. Most definitions adopt a generally positive valence, suggesting a gap with risk-heavy popular narratives. We show that, for researchers investigating where living meets machine, terminological precision is more than semantics and it can shape design, accountability, and public trust. This narrative review contributes a comparative taxonomy and a shared lexicon for reporting hybrid systems. Researchers are encouraged to clarify which sense of Hybrid-AI is intended (algorithmic fusion vs. human–AI ensemble), to specify agency locus and integration depth, and to adopt measures consistent with these conceptualizations. Such practices can reduce construct confusion, enhance cross-study comparability, and align design, safety, and regulatory expectations across domains.

## 1. Introduction

Walk through any robotics or AI conference and you can overhear a babel of terms: cyborg tissues, joint cognitive systems, neuro-symbolic hybrids, human–AI teaming. Each phrase refers to some combination of the living and the machine, but no one fully agrees on how tight the coupling is, or where agency and accountability lie. This definitional inconsistency hampers theory-building and comparability across studies. The issue is especially troublesome for the biomimetics community whose creations, straddling the border between living and machine, demand precise language to guide design and communication.

Early nomenclature set the stage for the definitional confusion we see today. In 1960, “cyborg” was introduced as “the exogenously extended organizational complex functioning as an integrated homeostatic system unconsciously” [1], anchoring the term in physiological integration—an image familiar to humans. Three decades later, the concept was broadened, “A cyborg is a cybernetic organism, a hybrid of machine and organism, a creature of social reality as well as a creature of fiction” [2], shifting attention from hardware to sociopolitical identity.

Contemporary scholarship fragments the definitional landscape further. AI researchers and defense technologists narrow Hybrid-AI to the fusion of symbolic and sub-symbolic methods, emphasizing representational synergy [3]. Information-systems researchers prefer hybrid intelligence—“the complementary strengths of human intelligence and AI so that they can perform better together than either could separately” [4]. Cognitive engineering offers joint cognitive systems: two or more cognitive systems, of which at least one includes a human, collaborating to keep a complex process in control [5].

Viewed side-by-side, these labels converge on the idea of coupling the living and the machine, but they diverge on agency, depth of integration, and moral valence. For designers crafting surgical cobots or wearable exoskins, typical artifacts in biomimetics, such ambiguity frustrates literature reviews and hampers science communication. Furthermore, for theorists measuring trust or workload, such unclear concepts can threaten construct validity and cross-study comparability. This lack of a shared lexicon is particularly evident in the case of Hybrid-AI. Although Hybrid-AI has a precise technical meaning that refers to the integration of symbolic and sub-symbolic AI methods, it is often misinterpreted in interdisciplinary and public discourse as describing human–AI collaboration or hybrid intelligence. This semantic drift blurs the boundary between machine–machine architectures and human–machine systems, creating confusion across technical, social-science, and policy domains. As a result, scholars and practitioners frequently talk past one another, which hinders comparative research and the coordination of design and regulatory frameworks. Therefore, exploring the key terminologies emphasized across disciplines and organizing them into coherent categories is an essential step toward conceptual clarity.

While a number of recent reviews have examined related themes, most remain anchored within a single discipline or conceptual niche. For example, studies in production management focus on hybrid intelligence as an optimization strategy [6]. Research in marketing, consumer research, and psychology investigates AI’s influence on consumer behavior through bibliometric analyses of theories and themes spanning cognition, social media analytics, and technology adoption [7,8]. Literature review work in journalism traces the rise of automated and algorithmic reporting, using thematic analyses to explore how artificial intelligence is applied across news production and storytelling [9]. Other efforts center on individual constructs such as AI literacy [10], automation [11], augmented intelligence [12], or human-in-the-loop systems [13]. Together, these reviews provide valuable but fragmented insights confined to particular domains. The present review builds on them by integrating studies from multiple databases across engineering and social-science disciplines, offering a more comprehensive view of both human-centered and technical perspectives. Owing to these differences in scope and methodology, our analysis departs from earlier discipline-bound syntheses and contributes a cross-field lexicon that identifies converging research fronts, terminological fault lines, and directions for future inquiry in hybrid-AI scholarship.

To map the definitional landscape, we assemble a curated corpus of 60 hybrid machine system (HMS) definitions spanning engineering, HCI, philosophy, and policy. We use HMS as a collective term encompassing any arrangement in which biological or human agents and artificial systems jointly contribute to perception, reasoning, or control. Within that family, Hybrid-AI refers specifically to architectures or teams in which symbolic and sub-symbolic intelligence are integrated with human cognition. We (1) identify semantic clusters, (2) extract cross-cutting dimensions such as agency locus and integration depth, and (3) propose a faceted taxonomy portable across disciplinary borders. Clarifying where concepts overlap—and where they do not—gives biomimetics researchers, and the broader hybrid-systems community, a common lexicon for cumulative science and responsible innovation. At the same time, we show that the language of “hybridity” has intensified in the wake of large-scale AI and biohybrid breakthroughs since 2020, making the present moment an inflection point for terminology.

The paper proceeds as follows. Section 2 situates HMS terminology within earlier work on cybernetics, distributed cognition, and neuro-symbolic AI. Section 3 details our data-collection and coding procedures. Section 4 presents clustering results and semantic dimensions. Section 5 discusses our findings and the implications, while Section 6 outlines limitations and boundary considerations.

## 2. Literature Review

Scholarly discourse on HMS has followed the same line. Three partially insulated traditions—cybernetics and the cyborg imaginary, distributed or joint cognition, and the newer conversation on hybrid or augmented intelligence—have matured in parallel, each bringing its own priorities and concepts. Subsequent empirical work often inherits these framings wholesale, rarely pausing to notice the alternatives that neighboring fields provide. The result is conceptual drift.

The first lineage is physiological and control-theoretic. Mid-twentieth-century cybernetics treated the body as a platform to be re-engineered for hostile environments, introducing the cyborg concept as an organism whose homeostatic functions extend beyond the skin through engineered add-ons [1]. In the very same year, another leading computer scientist proposed “man-computer symbiosis,” predicting “very close coupling between the human and the electronic members of the partnership” [14]—a parallel vision that centered cognitive, rather than physiological, integration. Together these twin 1960 visions framed technology as both body and thinking partner.

That same logic now drives research on bio-hybrid actuators, tissue–electrode interfaces, and neural prostheses. Over time, cultural layers accreted: later scholarship reframed the cyborg as a site of identity and boundary politics [2]. Others extended the idea further, arguing that humans are “natural-born cyborgs” who habitually offload cognition onto notebooks, calculators, and—now—smartphones [15]. Such work foreshadowed today’s wearable or tissue-integrated sensors that blur organismal and artefactual boundaries [16]. Even within biomimetics, therefore, cyborgs can signal anatomical fusion or sociopolitical hybridity, depending on which branch of the lineage an author draws upon. Recent demonstrations, such as the creation of biohybrid cardiac tissue seeded with nanoelectronics [17,18] or light-guided “cyborg insects” for search-and-rescue [19]—show how the term continues to evolve in laboratory practice between bioengineering jargon and layperson-friendly metaphor.

A second tradition emerges from cognitive systems engineering and the anthropology of practice. Field studies of navigation crews shifted cognition beyond the skull, embedding mental work in networks of people, artifacts, and representations [20]. Building on that idea, the notion of a joint cognitive system casts humans and artifacts as co-controllers of safety-critical processes [5]. Here the analytic unit is the functional ensemble—checklists, displays, spoken commands, tacit know-how. Agency is explicitly distributed, and research gravitates toward breakdowns, handovers, and resilience rather than toward anatomical fusion or representational synergy. Recent human-autonomy teaming surveys reveal the tension, with authors finding that “construct confusion” across human-automation teaming, human–robot interaction, and joint-cognitive systems studies creates difficulty in accumulating a cohesive scientific knowledge base [21]. Similar calls for terminological clarity are a key motivator for our research here.

The third and most recent genealogy stems from artificial-intelligence research and human–AI teaming. Information-systems scholars describe hybrid intelligence as the complementary strengths of humans and AI combining to achieve complex goals while learning from one another [4]. Others extended this view, arguing that hybridity lies not in blending algorithms but in orchestrating complementary competencies [22]. Industry white papers rebrand the same idea as augmented intelligence, insisting that machines enhance rather than replace human workers [23]. Providing an explicit example, the American Medical Association (AMA) explicitly frames AI as augmented intelligence that should support, not supplant clinical judgment [24], highlighting the normative stakes of word choice.

This stream is optimization-minded. Hybridity becomes a design variable: allocate each sub-task to whichever agent, statistical or biological, performs best, then tune for accuracy, speed, or cost. Thus, in this stream, debates focus less on system boundaries and more on workflow orchestration, explainability, and real-time calibration of decision authority. Yet the technical community also advances a different sense of hybridity—“Hybrid-AI” as neuro-symbolic integration [3]—where the fusion occurs within the algorithm rather than between human and machine. Distinguishing these two senses is obviously important because conflating them obscures which research problems are architectural in nature versus those that tread into the sociotechnical realm.

All three lineages insist on non-trivial coupling between human and machine. All treat that coupling as instrumental. Where they part company is the locus of agency, depth of integration, and moral valence. Cybernetic framings see agency as an embodied extension; distributed-cognition work views it as emergent across socio-technical ecologies; hybrid-intelligence research treats agency as a dial set task by task. Integration ranges from prosthetic fusion through representational coordination to interface-level orchestration. Normative stances vary from evolutionary adaptation through resilience seeking to performance optimization, each embedding tacit assumptions about autonomy, liability, and trust. These patterned disagreements matter. A biomimetics study framed in cyborg vocabulary invites questions about tissue compatibility and metabolic load; one written in joint-cognition terms will be judged on coordination and error recovery; a manuscript positioned around hybrid intelligence must speak to learning curves and decision metrics. Moreover, popular fascination with “droids” and “humanoids” amplifies the stakes: media headlines about Tesla’s Optimus robot or “ocean-going cyborg jellyfish” preload public expectations with both awe and anxiety, making terminological precision central to science communication [25].

Terminology becomes even more tangled when AI enters the scene. A human-in-the-loop system may be labeled “augmented intelligence” in human–computer interaction (HCI), “joint cognitive system” in human factors, or simply “AI” in psychology. Such variation obscures meaningful distinctions between non-AI automation, pure AI, and Hybrid-AI—the term we foreground here. Although Hybrid-AI has a precise technical meaning—the integration of symbolic and sub-symbolic methods for reasoning and learning [3]—public and interdisciplinary discourse often conflates hybrid with human–AI teaming. The word evokes flesh-and-circuit synergy, not architectural fusion. Consequently, many healthcare, education, and urban-governance projects describe collaborative decision support as Hybrid-AI despite employing only neural models plus a human operator. Conceptual drift happens. Definitions matter.

This review therefore asks:

RQ1: How do disciplines define Hybrid-AI and related human–machine systems concepts?

RQ2: Which Hybrid-AI system types dominate current studies?

RQ3: Which assumptions still need unpacking to enable cross-disciplinary research?

### Biomimetics: A Test-Bed for Terminological Collision

**Figure 1:**
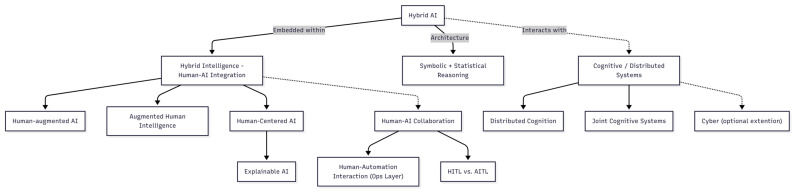
Concept Map. The concept map clarifies how Hybrid AI relates to adjacent concepts across three domains: algorithmic architecture, human–AI interaction, and cognitive/distributed systems. Positioned at the center, Hybrid AI is defined as the integration of symbolic reasoning and data-driven learning, an architectural approach that underpins intelligent agents. Extending from this are interaction models such as Human-Centered AI, Human–augmented AI, and Human-in-the-loop systems, which shift focus from algorithmic integration to shared agency, reciprocal learning, and decision-making dynamics. On the systems level, the map connects to Distributed Cognition, Joint Cognitive Systems, and Cyborgs, highlighting how intelligence can be distributed across human–machine assemblages. This system-level view treats human–machine systems as integrated cognitive units, where machines designed for specific functions join humans and tools in shared performance and distributed thinking. Note: Although the concept of “Cyborg” originated in human–machine interface technologies, its characteristics of human–machine integration and functional co-agency align with the perspective of distributed cognition, where cognition is modeled as a system-level process across humans and artifacts.

The study of biomimetics already showcases the complicated terminological map. A recent path-planning study combined multiple bio-inspired algorithms, dubbing the method a “hybrid swarm-intelligence algorithm” and reporting improved efficiency [26]. A 2025 investigation steered *Endebius florensis* beetles using backpack-mounted LEDs, labeling the creatures “cyborg insects” and “insect-machine hybrids” [19]. Earlier work wired microelectronics onto live jellyfish, producing a “biohybrid robotic jellyfish” that merged animal propulsion with on-board control [27,28]. Three papers, three labels—hybrid, cyborg, biohybrid (and even a mention of robots for good measure)—each pointing to living–machine fusion yet signaling different conceptualizations of it. The inconsistency underscores why a stable lexicon is urgent. Figure 1 organizes frequently conflated terms by theoretical focus (e.g., algorithmic architecture, human–AI interaction, and cognitive/distributed systems), clarifying where ‘Hybrid-AI’ (algorithmic) sits relative to ensemble notions such as hybrid intelligence or joint cognitive systems.

Recognizing the landscape, the remainder of this article treats genealogy as an analytic scaffold instead of historical anecdote. The contrasting assumptions about agency, integration, and normativity become coding dimensions applied to sixty verbatim definitions drawn from engineering, HCI, philosophy, and policy. The resulting comparative taxonomy aims to travel across disciplinary borders—and to give biomimetics researchers a clearer map for designing, evaluating, and naming the next generation of living-machine hybrids.

## 3. Methods

We undertook a narrative scoping review combined with a concept analysis to clarify how the terms AI, Hybrid-AI, and automation are defined and distinguished in human-centered contexts. Because the current debate is moving quickly, the search window was restricted to January 2020 through May 2025, and all database queries were executed in June 2025. Five bibliographic sources, PsycINFO, Scopus, the Communication & Mass-Media Complete index, IEEE Xplore, and LISA, returned ninety-one records. Citation chaining from key papers yielded thirty-four additional items, and manual checks contributed six more, giving a total of 131 hits. After removing ten duplicates and three records without minimally sufficient metadata, 118 items proceeded to full-text screening. Thirty-two were excluded for lacking definitional content or a human-facing relevance, leaving eighty-six reports that supplied sixty unique term-definition pairs.

The 2020–2025 corpus served primarily to identify high-frequency and widely discussed terms related to autonomy, AI, and Hybrid-AI, and to derive the analytic framework used for cross-disciplinary comparison. Focusing on this recent time period for term identification mitigated the risk of selecting older and/or out-of-use terms that would dilute our analysis. Because many papers in this corpus were exploratory and lacked explicit definitions, we subsequently conducted targeted follow-up searches without date limits to retrieve canonical or authoritative definitions of those terms. This two-stage strategy ensured both recency and conceptual completeness.

Eligibility hinged on five criteria applied in a single pass at full text: the work had to be peer-reviewed; it had to discuss definitions or conceptual boundaries of AI, Hybrid-AI, or automation; it had to address human use or impact rather than purely technical performance; it had to fall within the disciplines of human–computer interaction, human factors, psychology, communication, information or computer science (with occasional adjacent inclusions where definitional work was substantive); and it had to be published in English. Applying these rules produced a tractable but cross-disciplinary corpus that spans laboratory studies of joint cognitive systems, qualitative analyses of human–AI teaming, and technical proof-of-concepts for neuro-symbolic architectures used in healthcare or education. Table 1 details the coding rubric and decision rules. Notably, shared/dynamic agency (HM) required three explicit conditions (human execution channel, machine execution channel, and an articulated hand-off).

The unit of analysis for data extraction was the concept entry: a term, its verbatim definition, and its bibliographic source. Each concept was scored on three axes. We examined every definition to identify explicit or implicit cues for the three dimensions. The detailed coding scheme, including operational definitions and decision criteria, is summarized in Table 1. Agency locus captured where primary task initiative and authority reside and was encoded as human-led 0, shared 1, or machine-led = 2. Integration depth measured how tightly human and machine elements are coupled, ranging from loose 0, through moderate 1, to high 2. Normativity registered the overall valence of the framing, with positive +1, neutral 0, and negative −1. This coding exposed patterned disagreements that anecdotal reading tends to miss. For clustering, we collapsed −1/0/+1 to 0/1 to balance classes; the three-level distributions are reported descriptively. All entries were consolidated in a master sheet and coded numerically in a companion sheet (combined in Appendix A.1).

We also consulted contextual information when wording was ambiguous (e.g., the disciplinary domain of the source, such as HCI vs. control systems, stated assumptions about autonomy, or typical use cases) to ground coding decisions and avoid over-interpretation. Coders recorded brief evidence notes (e.g., keywords like approval/override, shared autonomy/teaming, autonomous/automatic) to document the rationale for each assignment. Two coders independently coded all definitions using the shared codebook. Inter-coder agreement (percent agreement) was high across the three dimensions: 91.7% (agency locus), 90.0% (integration depth), and 86.7% (normative orientation). Discrepancies were resolved through discussion to consensus, with the agreed labels constituting the final dataset for analysis.

To illuminate higher-order structure in the dataset, we submitted the three numerical codes to a k-means cluster analysis after standardizing the scale. Optimal k was determined by inspecting k = 3–5 and then maximizing silhouette width and retaining theoretical interpretability. Cluster selection was guided by two criteria: (1) the silhouette score, higher than 0.5 indicating moderate-to-good separation between clusters, and (2) a minimum cluster size threshold of 10 concepts to ensure interpretability and conceptual stability. Among all tested solutions, the k = 4 configuration maximized the silhouette coefficient (0.565) while meeting the size constraint, providing the most theoretically grounded and statistically robust solution.

**Figure 2:**
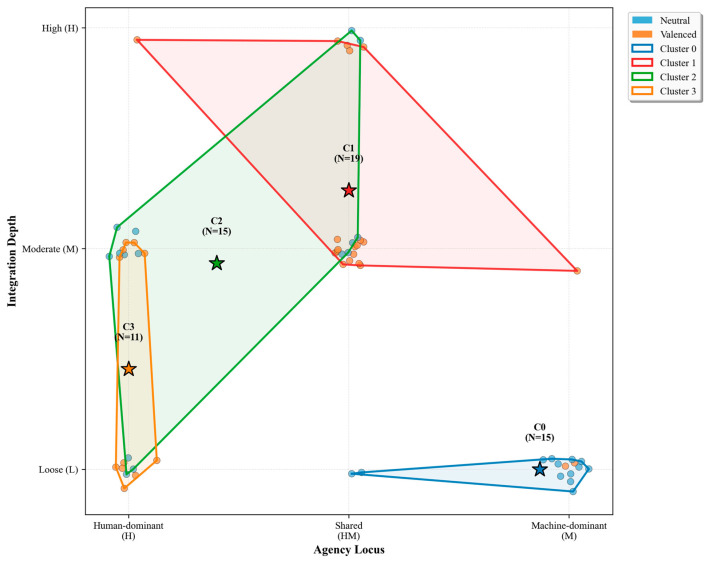
Visualization of K-means Clustering of 60 HMS-related Concepts. Each point is a term placed on Agency Locus (H–HM–M, *x*-axis) and Integration Depth (L–M–H, *y*-axis). Color encodes normative stance (Neutral vs. Valenced). Polygons depict cluster convex hulls and stars mark centroids; cluster IDs and sizes are labeled accordingly. Clustering used k-means on a three-dimensional matrix (Agency, Integration, Normative), with ordinal mappings H = −1, HM = 0, M = +1 and L = −1, M = 0, H = +1, and Valenced = 1, Neutral = 0. The solution differentiates (C0) machine-dominant, loosely coupled notions, (C1) shared-control, moderate–high coupling notions, (C2) interactive/distributed, moderate coupling notions, and (C3) human-dominant, loose–moderate coupling notions.

The resulting clusters map four regimes of hybridity. One is machine-led and loosely integrated, another is shared-agency with moderate-to-high integration and an optimistic tone, a third is human-led with medium coupling and a neutral stance, and the fourth is low-integration, human-centric, and positive. Cluster sizes were nineteen, fifteen, fifteen, and eleven, respectively, and centroids for agency, integration, and normativity are reported later in Table 2. Figure 2 visualizes the integration–agency landscape colored by these four clusters; the full coding rubric and membership roster appear in the Appendix A. Table 3 summarizes the distribution of Agency (H/HM/M), Integration (L/M/H), and Normativity (−1/0/+1) across the 60 concepts.

Taken together, the review supplies a disciplined vocabulary map rather than another list of buzzwords. By grounding definitions in explicit agency, coupling, and normative coordinates, the taxonomy can travel across disciplinary borders and—of special interest to Biomimetics readers, help designers of bio-hybrid actuators, cyborg insects, or hybrid swarm-intelligence algorithms decide what kind of “hybrid” they are really building.

The five-year window brackets what is arguably the fastest vocabulary acceleration in AI history, driven of course by the explosion of AI discourse after ChatGPT’s public release in December 2022 and preceding discussions around events like the introduction of AI-specific regulation in several nations. Restricting the search to 2020–2025 therefore captures the inflection point at which hybrid terminology migrated from specialist workshops into mainstream policy and press coverage. It also guards against retrofitted definitions applied to technologies that pre-date the current surge, ensuring the corpus reflects the language environment that current researchers and their lay audiences actually inhabit.

## 4. Results

The search conducted in June 2025 returned 131 records across five databases, citation chaining, and manual sources. After removing ten duplicates and three items with insufficient metadata, 118 records were assessed at full text. Thirty-two failed eligibility–typically because they offered no explicit definition or addressed purely technical performance, leaving 86 studies that anchored 60 distinct term-definition entries.

### 4.1. Concept Characteristics Before Clustering

Across the 60 entries, agency was most often described as shared between human and machine (41.7%), followed by human-led arrangements (35.0%) and a smaller contingent of machine-led systems (23.3%). Integration depth clustered around the middle of the scale: almost half of the concepts (48.3%) specified moderate coupling, 40.0% referred to loose tool-like links, and only 11.7% invoked high degrees of fusion. Framing was overwhelmingly neutral to optimistic—60.0% of definitions adopted an ambivalent or conditional tone, 38.3% were explicitly positive, and a single entry portrayed the technology in a negative light. Mean numerical scores across the three axes were 0.88 for agency, 0.72 for integration, and 0.53 for normativity, signaling a slight tilt toward shared control, moderate coupling, and favorable rhetoric (Table 3).

### 4.2. Cluster Landscape

Applying k-means to the three numeric axes yielded a four-group solution with a silhouette coefficient of 0.565. The clusters capture distinct regimes of hybridity. Machine-Led Low Integration Dominance (n = 15) aggregates concepts such as Hybrid-AI, neuro-symbolic architectures, and mixture-of-experts language models. These definitions emphasize algorithmic autonomy while keeping the human at arm’s length and adopt a largely neutral stance. Shared Collaborative Normative (n = 19) gathers terms like hybrid intelligence, mixed-initiative systems, and coactive design; here agency is balanced, integration ranges from moderate to high, and the tone is clearly upbeat. Human-Led Medium Integration (n = 15) encompasses AI-in-the-loop, joint cognitive systems, and decision-support tools, describing scenarios where humans retain primacy yet interact continuously with AI. Finally, Human-Centric Low Integration (n = 11) houses participatory AI, interactive machine learning, and RLHF, all framed as empowering technologies with light coupling and an explicitly positive ethos. Table 2 shows that Shared Collaborative Normative is the largest, followed by MachineLed Low Integration, HumanLed Medium, and HumanCentric Low.

Table 4 reports the centroid values for the three key dimensions across the four clusters. The listed top concepts illustrate how each cluster captures a distinct locus of meaning within the hybrid human–machine systems (HMS) landscape. Hybrid AI and Multi-Agent Systems in Cluster 0 embody the machine-led, low-integration end of the spectrum, where control and decision-making are primarily algorithmic and human input is minimal. Hybrid Intelligence and Shared Autonomy in Cluster 1 represent shared-control paradigms that emphasize mutual adaptation and complementary capabilities between humans and AI. AI-in-the-Loop and immersive technologies such as XR/VR in Cluster 2 mark a transition toward human-led yet technologically embedded arrangements, where interaction is continuous but the human remains central to control. Finally, Participatory AI and Augmented Decision Making in Cluster 3 anchor the human-centric pole, emphasizing inclusion, transparency, and empowerment in socio-technical systems. Figure 2 positions every concept along two coding dimensions: Agency Locus (*x*-axis) and Integration Depth (*y*-axis), with color indicating Normative Orientation (neutral vs. valenced). Cluster polygons outline the semantic regions identified by k-means analysis, and stars mark the centroids.

Cluster compositions reinforce these qualitative signatures. Eighty-seven percent of C0 entries assign primary control to the machine, whereas C3 is entirely human-centric. C1 alone contains high-integration definitions (26.3% of its items), echoing claims of deep symbiosis. Normative orientation also sorts cleanly: the two clusters dominated by human agency show the strongest positive framing, while neutral language prevails in C0 and C2. The plot reveals four distinct groupings: machine-led, loosely coupled systems (C0); shared-control, moderately to highly integrated systems (C1); interactive, human-led frameworks with moderate coupling (C2); and human-centric, low-integration models (C3)—together forming a continuum from technical autonomy to human-centered collaboration.

A working glossary of high-salience terms with their coded attributes and the full 60-item list is provided in the Appendix together with cluster assignments. CSV files underpinning all tables and figures accompany the data-availability statement.

### 4.3. Robustness Check

Alternative k values between three and six produced lower silhouette scores and less interpretable partitions; the four-cluster solution therefore represents both the best statistical fit and the clearest conceptual map.

## 5. Discussion

Our findings shed light on how different disciplines articulate the idea of “hybridity” at the living–machine interface. The literature reveals some consistent yet divergent interpretations that structure the definitional landscape. Answering our first research question, across computer science, HCI, biomimetics, psychology, communication, and information science, we observe two dominant senses of “hybrid”: (i) algorithmic fusion (e.g., Hybrid-AI, neuro-symbolic models) that is typically machine-led with loose coupling, and (ii) human–AI ensembles (e.g., hybrid intelligence, mixed-initiative systems) that emphasize shared or human-led agency with moderate coupling. Investigating our second research question (which types dominate), we find shared or dynamic arrangements are most common, while high-integration concepts remain rare. Our third research question (assumptions to unpack) reveals the complexity of the landscape because “Hybrid-AI” is applied to both algorithmic fusion and human–AI teaming.

Terminological ambiguity carries practical risks that extend beyond academic debate. When the same term is used inconsistently across disciplines, policymakers may ground regulations in divergent meanings, producing gaps in accountability or over-regulation of systems that differ in intent and architecture. In media coverage, such ambiguity could lead to public misperception, framing Hybrid-AI either as a threat to human autonomy or as a promise of effortless collaboration. Both frames are a distortion of how these systems actually function.

The present definitional landscape presents many discussion points, one of which is the valence asymmetry in our data. Of the sixty concept entries we analyzed, all but one are framed either neutrally or optimistically. In other words, 98 percent of the literature portrays hybrid systems as neutral, benign, promising technologies. This positive frame contrasts sharply with risk-oriented narratives that dominate popular media and policy debate, where job displacement, bias, and existential threat often take center stage. The gap is consequential, beyond venue effects, it can shape risk registers, regulatory scoping, and public expectations. Because our corpus draws primarily from design-forward, solution-oriented venues, the imbalance is understandable, but it still matters. When researchers speak the language of benefit while the public hears the language of harm, expectations (and even prospective regulation) can drift out of sync between scientists and society. Bridging that gap suggests small shifts in research practice, e.g., explicitly acknowledging foreseeable failure modes and normative assumptions in definitional work. Doing so keeps the scientific lexicon aligned with the realities of governance and public trust without dulling the optimism that motivates innovation.

Perhaps related to this valence pattern is the most consequential ambiguity in our data: two radically different senses of “Hybrid-AI.” On one side sit machine-meets-machine architectures such as neuro-symbolic pipelines, mixture-of-experts language models, hybrid swarm algorithms, etc. These entries gather in Cluster 0, characterized by machine-led agency and a general tone that is more technical and less moral or ethical. On the other side sits man-meets-machine teaming. Examples include mixed-initiative systems, coactive designs, and participatory learning loops. These populate Clusters 1 through 3 and carry the social and ethical burden of hybrid designs. An implication for researchers is that the risk of placing both families under the same label could obscure design requirements or evaluation criteria, and perhaps even the anticipated regulatory/accountability framework the technology will operate in. An algorithmic-fusion system typically (in some regulatory systems) may fall under software-safety regulation, whereas a human–AI ensemble may follow workplace or medical-device statutes. Label clarity therefore guides not only experimental design but also compliance pathways and insurance underwriting [24]. Therefore, the point is emphasized again that label clarity, while seemingly pedantic at times, smooths the development of new technologies in addition to aiding cumulative science.

Our single negative-valence concept in the entire dataset (algorithm aversion) is notable for being a behavioral construct rather than an architectural label. Algorithm aversion describes humans’ avoidance of deferring decisions to machine judgment (e.g., decision aids, autonomous agents, etc.). Negativity thus centers on adoption psychology rather than hybrid engineering per se. The implication is that design efforts aimed at transparency and trust repair (following errors) are important, even when designers are highly confident that systems will perform well. Some trust research show that explanations can flip aversion into appreciation within several interaction rounds [29,30], suggesting that the attitude effects are changeable rather than fixed.

Our results also show that the promise of true fusion—living and machine united into a seamless whole—remains more rhetoric than reality. Only seven of the 60 concepts register high integration. Most entries settle for a framing that suggests only moderate to loose coupling, even when framing elsewhere in the paper points to deeper integration. Thus, two futures are visible. One, a convergence trajectory, would see advances in AI and biomaterials pull today’s moderate designs toward tighter living-machine fusion. The other, an asymptote trajectory, would keep integration bounded by current constraints, consolidating shared-control systems rather than dissolving the boundary. The data signal that, for now, the field leans toward the latter. Tracking this trajectory is a key goal for future research investigating the ever-evolving conceptualizations of hybrid technologies.

Finally, the public imagination remains a wildcard. Sensational headlines about “cyborg soldiers” or “humanoid butlers” can overshadow careful laboratory language [25]. Because biomimetic prototypes often feature living material, mislabeling can provoke bio-ethical backlash—even when the work is benign. Clear messaging can pre-empt misunderstanding and foster informed debate, ensuring that innovation and societal trust grow in tandem.

Where, then, do the terms most familiar to Biomimetics readers reside on this map? Generally, most cluster in human-led or shared-agency regimes with moderate integration and neutral-to-positive framing. That placement aligns with the community’s historical emphasis on safety. It also implies a practical editorial guideline: when authors invoke “Hybrid-AI,” they should specify whether they mean algorithmic fusion or human–AI teaming, and they should state both the locus of agency and the depth of integration up front. Doing so will let reviewers and fellow researchers evaluate the newest technologies against the appropriate yardsticks.

Several limitations frame these findings. First, the scoping approach deliberately privileged human-centered, definitional work. Risk-heavy or policy-oriented studies are under-represented. Second, the three axes—agency, integration, normativity—compress nuance; shared autonomy, for example, covers a spectrum of task-allocation schemes that our coding treats as equivalent. Third, clustering relied on a binary normativity code to preserve statistical balance, whereas descriptive statistics retained the full three-level scale. Fourth, the four-cluster solution balanced silhouette fit with interpretability; finer subtypes undoubtedly exist. The taxonomy explains how concepts are used, not how systems perform; linking cluster membership to safety, workload, or error-recovery metrics remains future work. Finally, we searched and analyzed only English language sources and definitions.

## 6. Conclusions

Our comparative taxonomy offers a lexicon for reporting hybrid systems. Authors should consider stating which sense of Hybrid-AI is intended (algorithmic fusion vs. human–AI ensemble), specifying agency locus and integration depth, and choosing yardsticks that match the conceptualization. Doing so reduces construct confusion and improves cross-study comparability. More broadly, such practices may better align design, safety, and regulatory expectations across domains. As our research is a building step, we encourage future research on this topic so as to maintain an open, living glossary that records ongoing changes as the area evolves.

Taken together, the evidence suggests that the center of gravity in hybrid research is not full biological-machine fusion but competent, shared control. Algorithmic fusion—the technical heart of Hybrid-AI—remains largely value-silent, while ensemble practices bear the ethical weight. The practical ask for Biomimetics authors is simple: state which sense of “Hybrid-AI” you mean, and specify agency and integration explicitly. Precision in language is the first step toward designs that are safer, more comparable, and ultimately more useful across disciplines.

## Figures and Tables

**Table 1:** Coding Operational Sheet for Classifying HMS-related Concept.

Dimension	Code	Definition	Decision Criteria
Agency Locus	(H) Human-dominant	Human retains primary decision-making and control	Final approval, veto rights, or manual takeover is explicit.
	(HM) Shared/Dynamic	Control dynamically allocated or jointly shared between human and machine	Requires all three conditions: (a) Human execution channel explicit; (b) Machine execution explicit; (c) Shared control/handoff mechanism present.
	(M) Machine-dominant	Machine retains primary control; human mainly sets goals or monitors	Machine autonomy or automatic policy execution stated; no human override mentioned.
Integration Depth	(L) Loose coupling	Functional complementarity, low interdependence; systems run in parallel	No human actor mentioned in the definition or only for advisory/approval/offline interaction; human & machine loosely connected or separate.
	(M) Moderate coupling	Substantial information exchange and feedback loops without physiological embedding	Human actor is explicit, and coordination/feedback/interaction cues are present, but no physiological/biomechanical integration.
	(H) High coupling	Physiological, cognitive, or mechanical embedding and sustained, continuous interaction; human and machine form a unified operational system	Human and machine operate as a unified operational system; explicit reference to body/device-level integration & closed-loop interaction.
Normative Orientation	(1) Positive stance	Highlights benefits, efficiency, capability enhancement, and empowerment	Assumes trustworthiness, improved autonomy, or better outcomes.
	(−1) Negative stance	Highlights risks, harms, threats, or ethical concerns	Assumes loss of control, liability concerns, or erosion of trust.
	(0) Neutral stance	Purely descriptive or definitional, no explicit value judgment	Focuses on what it is, not what it does.
	(1,−1) Mixed stance	Contains both positive and negative normative signals	Reflects uncertain trade-offs, dual-use nature, or socio-ethical complexity.

**Table 2:** Cluster Sizes & Composition.

Cluster	Label	Size (n)	Percentage
C0	Machine-Led Low Integration	15	25.00%
C1	Shared Collaborative Normative	19	31.70%
C2	Human-Led Medium Integration	15	25.00%
C3	Human-Centric Low Integration	11	18.30%
Total		60	100%

**Table 3:** Distribution across agency, integration, and normativity (*n* = 60).

Dimension	Level/Value	n	% of Total
Agency	Human-led	21	35.0%
	Shared	25	41.7%
	Machine-led	14	23.3%
Integration	Low	24	40.0%
	Medium	29	48.3%
	High	7	11.7%
Normativity	Negative	1	1.7%
	Neutral	36	60.0%
	Positive	23	38.3%
Means	Agency = 0.88 Integration = 0.72 Normativity = 0.53		

**Table 4:** Cluster Centroids & Top Concept.

Cluster	Label	Agency Centroid	Integration Centroid	Normativity Centroid	Top Concepts
Cluster 0	Machine-Led Low Integration	1.87	0.00	0.13	Hybrid-AI, Neuro-Symbolic AI, Multi-Agent System
Cluster 1	Shared Collaborative Normative	1.00	1.26	1.00	Hybrid Intelligence, Adjustable Autonomy, Shared Autonomy
Cluster 2	Human-Led Medium Integration	0.40	0.93	0.00	AI-in-the-Loop (AITL), Immersive Technology (XR), Virtual Reality (VR)
Cluster 3	Human-Centric Low Integration	0.00	0.45	1.00	Participatory AI, Augmented Decision Making

## Data Availability

All data supporting the findings of this study are provided in the Appendix A.

## Abbreviations

The following abbreviations are used in this manuscript:

AI	Artificial Intelligence
HMS	Hybrid Machine Systems
HCI	Human–computer Interaction
BCI	Brain Computer Interface
HITL	Human in the Loop
AITL	AI in the Loop
JCS	Joint-Cognitive Systems

## Appendix A

### Appendix A.1

**Table A1:** Terms, Definitions, and Axis.

Term	Standard Definition	Reference Source	Agency Locus	Integration Depth	Normativity
Hybrid-AI	Combines heterogeneous AI paradigms (e.g., symbolic reasoning and neural learning) so their complementary strengths yield more robust, generalizable systems.	“Hybrid AI is defined as the combination of reasoning on symbolic knowledge with machine learning from data (objects) embodied in an intelligent agent. Hybrid AI is part of systems of Hybrid Intelligence (HI), where intelligent agents learn and reason and interact with humans and other agents in relation to the environment that they are situated in. Intelligent agents need to acquire awareness about themselves, their context and users (both human and other agents” [31]	M	L	0
Hybrid Intelligence	The combined intelligence of humans and AI systems, leveraging complementary strengths.	“A hybrid AI approach that integrates data-driven machine learning with domain knowledge, optimization, and reasoning” [32].	HM	Ms	1
Neuro-Symbolic AI	Integrates neural representation learning with symbolic knowledge and reasoning to improve interpretability, data efficiency, and generalization.	“The ability to achieve complex goals by combining human and artificial intelligence, thereby reaching superior results to those each of them could have accomplished separately, and continuously improve by learning from each other” [4].	M	L	0
Multi-Agent System	A setting where multiple autonomous agents interact: cooperatively or competitively: to solve tasks that exceed the capability of any single agent.	“The AI-human hybrid outperforms its human-only or LLM-only counterpart” [33].	M	L	0
Agentic AI (LLM Agents & Orchestration)	Large language model-based agents plan, act, and coordinate tools or sub-agents to pursue goals, often under an orchestration framework for reliability and control.	“Neuro-Symbolic AI is defined as the subfield of artificial intelligence (AI) that combines neural and symbolic approaches” [34].	M	L	0
Mixture-of-Experts (MoE) LLMs	Sparse architectures route tokens to specialized expert sub-networks, increasing capacity without proportional compute at inference.	“A Multi-Agent System is characterized by properties including flexible autonomy, reactivity, pro-activeness, social ability, the distributable nature of agents, the possibility of emergent behavior, and the fault tolerance [35].	M	L	0
Human-in-the-Loop (HITL)	A system where AI performs inference and decision-making, and humans intervene when necessary to provide corrections, feedback, supervision, or domain knowledge.	“A Multi-Agent System (MAS) is an extension of the agent technology where a group of loosely connected autonomous agents act in an environment to achieve a common goal” [36].	M	L	0
AI-in-the-Loop (AITL)	A system where humans make the final decisions, and AI assists by offering perception, inference, suggestions, or action support.	“Multi-agent systems (MAS) are a fast developing information technology, where a number of intelligent agents, representing the real world parties, co-operate or compete to reach the desired objectives designed by their owners” [37].	H	M	0
Human on the loop	Humans monitor autonomous operations and intervene or override when needed, maintaining ultimate authority without micromanaging each action.	“Agentic AI systems represent an emergent class of intelligent architectures in which multiple specialized agents collaborate to achieve complex, high-level objectives utilizing collaborative reasoning and multi-step planning” [38].	H	L	0
Adjustable Autonomy	The locus of control shifts dynamically between human and agent based on context, competence, and risk to balance safety and efficiency.	“The MoE framework is based on a simple yet powerful idea: different parts of a model, known as experts, specialize in different tasks or aspects of the data” [39].	HM	M	1
Shared Autonomy	Human intent and autonomous assistance are blended in real time so the system helps without seizing full control.	“Human-in-the-Loop is a configuration where humans perform “supervisory control in computational systems’ operations” requiring “human feedback and responsibility for performance management, exception handling and improvement” [40].	HM	M	1
Mixed-Initiative Systems	Both human and AI proactively propose actions or plans, negotiating initiative and control throughout the task.	“AI systems drive the inference and decision-making process, but humans intervene to provide corrections and supervision” [41].	HM	M	1
Coactive Design	Design emphasizes interdependence: agents are built to anticipate, expose, and adapt to human needs so joint activity remains fluent.	“Humans make the ultimate decisions, while AI systems assist with perception, inference, and action” [41].	HM	M	1
Human–AI Collaboration	Humans and AI work together toward shared outcomes when AI typically serves as a tool or assistant.	Humans on the loop positions humans as supervisors of automated systems. Instead of being directly involved in every decision (as HITL), humans monitor processes and intervene only when necessary, typically when anomalies or edge cases arise [42].	HM	M	1
Human–AI Teaming	Humans and AI function as interdependent teammates, sharing situation awareness, coordinating roles, and adapting to each other’s strengths.	“Provides an autonomous system with a variable autonomy in which its operators have the options to work in different autonomy states and change its level of autonomy while the system operates” [43].	HM	M	1
Human–AI Co-Creation	Creative workflows weave human intent with generative AI capabilities to explore options, iterate, and refine outcomes together.	“Systems that share control with the human user” [44].	HM	M	1
Human–AI Complementarity	Integrating human strengths (context understanding, ethical reasoning, sensemaking) with AI strengths (scale, speed, pattern recognition) to achieve joint performance gains.	“In shared autonomy, a user and autonomous system work together to achieve shared goals” [45].	HM	M	1
Participatory AI	Stakeholders help specify objectives, constraints, and impacts of AI systems, embedding participation across design and governance.	“Mixed-initiative refers to a flexible interaction strategy, where each agent can contribute to the task what it does best” [46].	H	L	1
Shared Mental Models	Team members maintain compatible representations of goals, roles, and system state, enabling coordination with minimal overhead.	This definition explicitly contrasts with adaptive systems (agent responds without intervention) and adaptable systems (agent suggests pre-programmed actions), highlighting its core principle of collaborative human-agent decision-making. The article further describes this model as capturing “the advantage of adaptive and adaptable processes without sacrificing the human’s decision authority” [47].	HM	M	1
Co-Adaptive Systems	Humans and AI adapt to each other over time, updating strategies, interfaces, or models to improve joint performance.	“Mixed-initiative human–robot teams” [48].	HM	M	0
Interactive Machine Learning (IML)	Humans iteratively provide labels, features, or constraints while observing model updates, closing the loop between learning and use.	“The term ‘coactive design’ was coined as a way of characterizing an approach to the design of HRI that takes interdependence as the central organizing principle among people and robots working together in joint activity” [49].	H	M	1
Reinforcement Learning from Human Feedback (RLHF)	Policies are optimized against human preference signals so model behavior aligns with human values and instructions.	A collaborative system where humans and AI work together on a task. This involves integration, interaction, or collaboration to leverage complementary capabilities [50].	H	M	1
Preference-Based Learning	Agents infer a preference ordering over trajectories or outcomes from comparisons or feedback rather than explicit rewards.	“Adaptable automation together with effective human-factored interface designs are key to enabling human-automation teaming” [51].	H	M	1
Decision Support Systems (DSS)	Interactive information systems that synthesize data, models, and user judgment to aid: not replace: managerial decision-making.	“Human–AI teaming focuses on interactions between human and AI teammates where a team must jointly accomplish collaborative tasks with shared goals” [52].	H	L	0
Adaptive Automation	Automation level is adjusted in response to operator state or task demands, mitigating overload while preserving engagement.	Humans and generative AI systems jointly shape creative outputs, with both actively manipulating and refining the artifacts under development [53].	HM	M	1
Augmented Decision Making	AI and analytics enhance human judgment by revealing patterns, forecasts, and counterfactuals, enabling better and more informed decision-making while humans retain final authority.	Combining the distinct, non-overlapping capabilities of humans and artificial intelligence to “achieve superior results in comparison to the isolated entities operating independently” [54].	H	L	1
Human-Augmented AI	AI systems where human knowledge, feedback, or demonstrations are integrated into training and deployment, enabling models to perform more reliably and effectively in real-world contexts.	Participatory AI can be defined as an approach that empowers end users to directly interact with and guide AI systems to create solutions for their specific needs [55].	H	L	1
Human Factors (in AI)	A research area focusing on how humans perceive, interact with, and are influenced by AI systems.	“Shared mental models are shared knowledge structures allowing team members to draw on their own well-structured knowledge as a basis for selecting actions that are consistent and coordinated with those of their teammates” [56].	H	L	0
Explainable AI (XAI)	Techniques and methods that make AI systems’ decisions, reasoning processes, and outputs understandable to humans, improving transparency, trust, and accountability without necessarily reducing performance.	“Both a human user and a machine should be able to adapt to the other through experiencing the interactions occurring between them” [57].	M	L	1
Human-Centered AI (HCAI)	An approach that keeps human values, control, and usability at the forefront of AI design, deployment, and governance.	Sub-systems or agents undergo “reciprocal selective pressures and adaptations” driven by “reciprocal adaptations between and within socio-economic and natural systems” [58].	H	M	1
Augmented Intelligence	Positions AI as an amplifier of human abilities rather than a replacement, emphasizing complementary roles and oversight.	Interactive Machine Learning (IML) is defined as a process where “model gets updated immediately in response to user input), focused (only a particular aspect of the model is updated), and incremental (the magnitude of the update is small). It also “allows users to interactively examine the impact of their actions and adapt subsequent inputs to obtain desired behaviors” [55].	H	L	1
Augmented Cognition	Uses AI and adaptive systems to support or enhance cognitive processes (e.g., attention, memory, decision-making) via real-time feedback and adaptive interfaces.	Reinforcement Learning from Human Feedback (RLHF) is a training procedure whose “only essential steps are human feedback data collection, preference modeling, and RLHF training” [59].	H	M	1
Human–robot Interaction (HRI)	Studies and designs two-way human–robot engagement: from physical collaboration to social communication: for safe, efficient, and intuitive teamwork.	Preference-Based Learning is a method that learns desired reward functions by asking humans for their relative preferences between two sample trajectories instead of requesting demonstrations or numerical reward assignments [60,61].	HM	M	0
Collaborative Robots (Cobots)	Robots designed to operate in close proximity with people, using safety-rated control and interaction modes to share workspaces.	“A decision support system (DSS) is defined as an interactive computer-based information system that is designed to support solutions on decision problems” [62].	HM	M	1, −1
Humanoid/Social Robot	Robots with humanlike form or social behaviors that support communication, instruction, or companionship.	Adaptive automation is defined as “the dynamic allocation of control of system functions to a human operator and/or computer over time with the purpose of optimizing overall system performance” [63].	M	M	1, −1
Bionic Human	A human enhanced or restored by integrating artificial components, such as prosthetics, implants, or wearable robotics, that augment, restore, or replace physiological and sensory functions, enabling improved mobility, strength, or perception.	The use of AI insights to “enhance managerial decisions” by analyzing large datasets, detecting patterns, and providing recommendations, while humans retain responsibility for responsibility over decision and action selection [64].	HM	H	1, −1
Wearable Robotics	A broad class of robotic devices worn on the body that assist, augment, or restore human movement, strength, or sensory functions.	“Human-augmented AI refers to AI systems that are trained by humans and continuously improve their performance based on human input” [65].	HM	H	1, −1
Powered exoskeleton	A wearable robotic framework with motorized actuators that assist, augment, or restore human movement and strength through real-time sensorimotor synchronization, used in rehabilitation, industry, military, and mobility enhancement.	“Human Factors as “a very broad discipline that encompasses human interaction in all its task-oriented forms” and addresses “traditional human-factors problems such as trust in automation” for the AI-specific angle [66].	HM	H	1, −1
Embodied AI	Intelligence emerges through an agent’s body interacting with the world, coupling perception, action, and learning.	“Experiences, behavioral preferences, decision-making styles,” along with their susceptibility to biases of end users constitute human factors impacting AI usage [67].	M	L	0
Brain–Computer Interface (BCI)	A technology that enables direct communication between the human brain and external devices, allowing AI systems to read, interpret, or influence neural activity for purposes such as control, augmentation, or rehabilitation.	Al system capable of explaining how it obtained a specific solution (e.g., a classification or detection outcome) and answering “wh” questions (such as “why”, “when”, “where”, etc.). This capability is absent in traditional Al models [68].	H	H	1, −1
Immersive Technology (XR)	An umbrella for virtual, augmented, and mixed reality that blends real and synthetic stimuli to expand perception and action.	“HCAI is an approach that seeks to position humans at the center of the AI lifecycle, improving human performance reliably, safely, and trustworthily by augmenting human capabilities rather than replacing them, with a focus on human well-being, values, and agency [69,70].	H	M	0
Virtual Reality (VR)	Computer-generated environments create a sense of presence that supports training, simulation, and embodied experimentation.	A human-centric AI mechanism is an approach that centers human needs and values by ensuring human control, usability, explainability, ethical safeguards, and user involvement throughout AI development [70].	H	M	0
Augmented Reality (AR)	Digital content is registered onto the physical world in real time to guide perception, action, or collaboration.	“Augmented Intelligence is defined as “a situation where the Human–AI combination/system performs better than the human working alone” [50].	H	M	0
Cyber-Physical Systems (CPS)	Systems that tightly integrate computation, networking, and physical processes through sensing, communication, and control loops, enabling real-time monitoring, coordination, and automation across diverse domains.	“AI systems augment human intelligence... AI systems tend to extend or amplify human capabilities by providing support systems such as predictive analytics rather than replacing them, resulting in augmented (human) intelligence” [65].	M	L	0
AI Use Case	A specific scenario where AI is applied to solve a problem or address a particular need, such as fraud detection in finance, digital twin cities, medical diagnosis, or autonomous driving.	“Augmented cognition is a form of human-systems interaction in which a tight coupling between user and computer is achieved via physiological and neurophysiological sensing of a user’s cognitive state” [71].	M	L	0
Distributed Cognition	Cognitive processes are spread across people, artifacts, and environments rather than contained solely in individual minds.	HRI is a field reviewing “the current status” and challenges of interactions between humans and robots [72].	HM	H	0
Joint Cognitive Systems	Human and machine form a single cognitive unit whose resilience depends on coordination, feedback, and graceful extensibility.	“Collaborative robots (cobots) are industrial robots designed to work alongside humans with the ability to physically interact with a human within a collaborative workspace without needing additional safety structures such as fences” [73].	HM	H	0
Sociotechnical Systems (STS)	Sociotechnical System views humans, technologies, and organizations as interdependent components, emphasizing the joint optimization of social and technical factors for effective outcomes.	“Collaborative robots are such robots that are designed to work along their human counterparts and share the same working space as co-workers” [74].	HM	L	0
AI-Mediated Communication (HMC)	AI transforms how people create, filter, and interpret messages, introducing new norms and risks in mediated interaction.	Humanoid/Social Robots are designed to “help through advanced interaction driven by user needs (e.g., tutoring, physical therapy, daily life assistance, emotional expression) via multimodal interfaces (speech, gestures, and input devices)” [75].	HM	M	0
Cyborg	A human-technology assemblage that maintains homeostasis through integrated biological and artificial components.	A humanoid/social robot is designed to “look and behave like a human” and can “more meaningfully engage consumers on a social level” than traditional self-service technologies [76].	HM	H	1, −1
Post-human Assemblage	A post-humanist concept describing networks of humans, AI, technologies, and environments where agency, identity, and meaning are co-constructed and distributed beyond the human.	“You may know someone with an artificial leg or arm. You may know someone with a hearing aid. Even if you don’t know anyone like that, it is likely that you or someone you know wears glasses. These people are allbionichumans. Any artificial-that is, man-made-part or device that is used on a person is called a prosthesis” [77].	HM	M	0
Human–AI Coevolution	Humans and AI systems evolve together over time, with mutual influence on capabilities, behaviors, and decision-making frameworks, leading to adaptive changes on both sides.	“Sense, and responsively act, in concert with their wearer”; “encompassing the human body and its surroundings as their environment” [78].	HM	M	1, −1
Human–computer Interaction (HCI)	A discipline that studies and designs interactive computing to fit human capabilities, needs, and values.	“The exoskeleton is an external structural mechanism with joints and links corresponding to those of the human body” [79].	H	M	0
Algorithm Aversion	People discount algorithms after observing small mistakes, preferring human judgment even when the algorithm is statistically superior.	“Embodied AI integrates “traditional intelligence concepts from vision, language, and reasoning into an artificial embodiment” to solve tasks in virtual environments [80].	H	L	−1
Algorithm Appreciation	People sometimes weigh algorithmic advice more than human advice, particularly for objective tasks and aggregated predictions.	“Embodied AI is designed to determine whether agents can display intelligence that is not just limited to solving abstract problems in a virtual environment (cyber space), but that is also capable of navigating the complexity and unpredictability of the physical world” [81].	H	L	1
Autonomous System	A physical or digital system capable of performing tasks or making decisions independently using its perception, reasoning, and control capabilities, with minimal or no human intervention.	Brain–computer interfaces (BCI) are systems that “activate electronic or mechanical devices with brain activity alone” [82].	M	L	0
Transformative AI (TAI)	AI systems defined by their consequences, capable of driving societal transitions comparable to or greater than the agricultural or industrial revolution.	Immersive technology is defined as technologies like augmented reality (AR) and virtual reality (VR) that provide customers with “interactivity, visual behavior, and immersive experience” [83].	M	L	1
General-purpose AI (GPAI)	General-purpose AI refers to AI Systems that have a wide range of possible uses, both intended and unintended by the developers. They can be applied to many different tasks in various fields, often without substantial modification and fine-tuning.	“VR is defined as “the use of computer-generated 3D environment, that the user can navigate and interact with, resulting in real-time simulation of one or more of the user’s five senses,” characterized by visualization, immersion, and interactivity [84].	M	L	0
Conversational AI	Conversational AI is an AI system designed to interact with humans through natural language processing and generation.	VR specifically offers “a realistic computer-generated immersive environment” that allows user perception of senses like vision, hearing, and touch [83].	H	M	0
Generative AI	AI systems that create new content: such as text, images, audio, video, or code: by learning patterns from data and generating original, context-relevant outputs.	AR is defined as “the enhancement of a real-world environment using layers of computer-generated images through a device” [84].	HM	L	0
Categorical Variable Encoding: Agency Locus: Human-dominant (H) → 0, Shared (HM) → 1, Machine-dominant (M) → 2 Integration Depth: Low (L) → 0, Moderate (M) → 1, High (H) → 2 Normativity: Neutral (“0”) → 0, Value-oriented (“1”, “-1”, “1,-1”) → 1					

### Appendix A.2

**Table A2:** Concept Distribution Across Four Clusters.

Machine-Led Low Integration Dominance	Shared Collaborative Normative	Human-Led Medium Integration	Human-Centric Low Integration
AI Use Case	Adaptive Automation	AI-Mediated Communication (HMC)	Algorithm Appreciation
Agentic AI (LLM Agents & Orchestration)	Adjustable Autonomy	AI-in-the-Loop (AITL)	Algorithm Aversion
Autonomous System	Bionic Human	Augmented Reality (AR)	Augmented Cognition
Cyber-Physical Systems (CPS)	Brain–Computer Interface (BCI)	Co-Adaptive Systems	Augmented Decision Making
Embodied AI	Coactive Design	Conversational AI	Augmented Intelligence
Explainable AI (XAI)	Collaborative Robots (Cobots)	Decision Support Systems (DSS)	Human-Augmented AI
General-purpose AI (GPAI)	Cyborg	Distributed Cognition	Human-Centered Artificial Intelligence (HCAI)
Generative AI	Human–AI Co-Creation	Human Factors (in AI)	Interactive Machine Learning (IML)
Human-in-the-Loop (HITL)	Human–AI Coevolution	Human on the loop	Participatory AI
Hybrid AI	Human–AI Collaboration	Human–computer Interaction (HCI)	Preference-Based Learning
Mixture-of-Experts (MoE) LLMs	Human–AI Complementarity	Human–robot Interaction (HRI)	Reinforcement Learning from Human Feedback (RLHF)
Multi-Agent System	Human–AI Teaming	Immersive Technology (XR)	
Neuro-Symbolic AI	Humanoid/Social Robot	Joint Cognitive Systems	
Sociotechnical Systems (STS)	Hybrid Intelligence	Post-human Assemblage	
Transformative AI (TAI)	Mixed-Initiative Systems	Virtual Reality (VR)	
	Powered exoskeleton		
	Shared Autonomy		
	Shared Mental Models		
	Wearable Robotics		
